# A Major Quantitative Trait Loci Cluster Controlling Three Components of Yield and Plant Height Identified on Chromosome 4B of Common Wheat

**DOI:** 10.3389/fpls.2021.799520

**Published:** 2022-01-11

**Authors:** Shaozhe Wen, Minghu Zhang, Keling Tu, Chaofeng Fan, Shuai Tian, Chan Bi, Zelin Chen, Huanhuan Zhao, Chaoxiong Wei, Xintian Shi, Jiazheng Yu, Qixin Sun, Mingshan You

**Affiliations:** ^1^State Key Laboratory for Agrobiotechnology, Key Laboratory of Crop Heterosis and Utilization (MOE), Beijing Key Laboratory of Crop Genetic Improvement, China Agricultural University, Beijing, China; ^2^Department of Plant Genetics and Breeding, College of Agriculture and Biotechnology, China Agricultural University, Beijing Innovation Center for Seed Technology (MOA), Beijing Key Laboratory of Crop Genetic Improvement, Beijing, China; ^3^Key Laboratory of Crop Germplasm Resources and Utilization, Ministry of Agriculture, National Key Facility for Crop Gene Resources and Genetic Improvement, Institute of Crop Sciences, Chinese Academy of Agricultural Sciences, Beijing, China

**Keywords:** common wheat, three components of yield, plant height, QTL cluster, fine mapping

## Abstract

Wheat yield is not only affected by three components of yield, but also affected by plant height (PH). Identification and utilization of the quantitative trait loci (QTL) controlling these four traits is vitally important for breeding high-yielding wheat varieties. In this work, we conducted a QTL analysis using the recombinant inbred lines (RILs) derived from a cross between two winter wheat varieties of China, “Nongda981” (ND981) and “Nongda3097” (ND3097), exhibiting significant differences in spike number per unit area (SN), grain number per spike (GNS), thousand grain weight (TGW), and PH. A total of 11 environmentally stable QTL for these four traits were detected. Among them, four major and stable QTLs (*QSn.cau-4B-1.1*, *QGns.cau*-*4B-1*, *QTgw.cau-4B-1.1*, and *QPh.cau-4B-1.2*) explaining the highest phenotypic variance for SN, GNS, TGW, and PH, respectively, were mapped on the same genomic region of chromosome 4B and were considered a QTL cluster. The QTL cluster spanned a genetic distance of about 12.3 cM, corresponding to a physical distance of about 8.7 Mb. Then, the residual heterozygous line (RHL) was used for fine mapping of the QTL cluster. Finally, *QSn.cau-4B-1.1*, *QGns.cau*-*4B-1*, and *QPh.cau-4B-1.2* were colocated to the physical interval of about 1.4 Mb containing 31 annotated high confidence genes. *QTgw.cau-4B-1.1* was divided into two linked QTL with opposite effects. The elite NILs of the QTL cluster increased SN and PH by 55.71–74.82% and 14.73–23.54%, respectively, and increased GNS and TGW by 29.72–37.26% and 5.81–11.24% in two environments. Collectively, the QTL cluster for SN, GNS, TGW, and PH provides a theoretical basis for improving wheat yield, and the fine-mapping result will be beneficial for marker-assisted selection and candidate genes cloning.

## Introduction

As one of the most important food crops in the world, common wheat (*Triticum aestivum* L.) is essential in satisfying human demand for calories and guaranteeing food security. In the last two decades, the rapid growth of the human population and the shrinking of available arable land have posed great challenges to the food supply (Lobell et al., 2011; Tilman et al., 2011). Therefore, high yield is still one of the main goals of wheat breeding programs. In addition to appropriate field management, identification and utilization of superior alleles for yield-related traits is one of the most effective ways to increase yield.

Like other cereal crops, wheat yield is a complex quantitative trait determined by three components (Simmonds et al., 2014), and is influenced by heredity and environment. Among these three components of yield, there have been many studies on quantitative trait loci (QTL) mapping for thousand grain weight (TGW) due to phenotypic stability and high heritability (Kuchel et al., 2007). A lot of QTL for TGW have been detected on majority 21 wheat chromosomes (Zhang et al., 2010; Gao et al., 2015; Sun et al., 2017; Zhai et al., 2018). TGW can be broken down into individual components including grain configuration parameters (grain length, grain width, grain thickness, and grain area) and grain filling characteristics (rate and duration) (Gegas et al., 2010; Zanke et al., 2015; Guan et al., 2019). So far, several homologous genes that control grain size in rice have been cloned, providing better insights into the genetic basis of grain size in wheat (Nadolska-Orczyk et al., 2017), such as *Grain Size 5* (*TaGS5-3A*) (Ma et al., 2016), *Grain Weight 2* (*TaGW2-A1*) (Simmonds et al., 2016), *Grain Length 3* (*TaGL3-5A*) (Yang et al., 2019), and *Cytokinin Oxidase 6* (*TaCKX6-D1*) (Zhang et al., 2012).

Grain number per spike (GNS), one of three components of yield, is determined by the number of fertile spikelets and florets per spike (Sreenivasulu and Schnurbusch, 2012), and is positively correlated with spike length (SL). Previous studies have identified a large number of QTL for spike-related traits on all wheat chromosomes by using linkage analysis and association analysis, including total spikelet number per spike (TSS), fertile spikelet number per spike (FSS), sterile spikelet number per spike (SSN), and SL (Li et al., 2007; Ma et al., 2007; Cui et al., 2012; Zhai et al., 2016; Guo Z. et al., 2018; Yao et al., 2019). To date, several genes controlling GNS have been isolated and characterized using a homology-based approach, such as *cytokinin oxidase 2.1* (*TaCKX2.1*), *cytokinin oxidase 2.2* (*TaCKX2.2*) (Zhang et al., 2011), *MONOCULM 1* (*TaMOC1-7A*) (Zhang et al., 2015), and *transcript elongation factor* (*TaTEF-7A*) (Zheng et al., 2014).

Unlike TGW and GNS with phenotypic stability, spike number per unit area (SN) is easily affected by environmental factors. For example, planting density (Nerson, 1980; Kondić et al., 2017), fertilizer management (Xu et al., 2014), and light condition (Evers et al., 2006). Therefore, few QTLs for SN have been detected at present (Marza et al., 2006; Heidari et al., 2011). Previous researches showed that many QTLs controlling tiller numbers per plant (TNPP) have been detected on multiple wheat chromosomes (Li et al., 2010; Naruoka et al., 2011; Hu et al., 2017; Liu J. et al., 2018; Wang et al., 2018). Moreover, four genes for TNPP have been cloned using mutants (Kuraparthy et al., 2006; Kebrom et al., 2012; Zhang et al., 2013b). Since the tillers do not all develop into effective spikes in the general field condition of wheat production, the increase in TNPP does not necessarily increase SN and ultimately improve yield. Thus, it is crucial to dissect the molecular basis of SN for improving wheat yield.

Plant height (PH) is another essential trait related to yield. The “Green Revolution” of crops in the 1960s, characterized by reduced PH, greatly increased crop yield. To date, 25 semidwarfing or dwarfing genes have been reported in wheat (Mo et al., 2018). Among them, *Rht1* (*Rht-B1b*), *Rht2* (*Rht-D1b*), and *Rht8* are three the most widely utilized dwarfing genes in wheat breeding worldwide (Ellis et al., 2002; Zhang et al., 2006; Tian et al., 2017). The *Rht1* and *Rht2* have been proved to have a negative effect on GNS and TGW (Zhang et al., 2013a), while the *Rht8* does not (Zhai et al., 2016; Tian et al., 2017). Therefore, identified genetic loci associated with PH and yield components can provide an in-depth understanding of their genetic relationships (Guan et al., 2018; Chen et al., 2020).

In the work reported here, we developed a recombinant inbred lines (RILs) population derived from a cross between Nongda981 (ND981) and Nongda3097 (ND3097) to perform QTL analysis on three components of yield and PH, with the purpose to dissect the genetic basis of these four traits. In addition, to validate the additive genetic effect of the QTL cluster on chromosome arm 4BS and further narrow the candidate region, the advanced separated population constructed by residual heterozygous line (RHL) were used for screening recombinants and subsequent Student’s *t-*test. Finally, three QTLs (*QSn.cau-4B-1.1*, *QGns.cau*-*4B-1*, and *QPh.cau-4B-1.2*) controlling SN, GNS, TGW, and PH, respectively, were delimited to a physical interval of about 1.4 Mb by the insertion/deletion (InDel) markers *INDEL(4B)27-1* and *INDEL(4B)29-1*, and two linked QTL (*QTgw.cau-4B-1.1^ND981^* and *QTgw.cau-4B-1.1*^ND3097^) for TGW with opposite effects were narrowed to the interval of approximately 12.3 Mb, which lays a foundation for marker-assisted selection and map-based cloning.

## Materials and Methods

### Plant Materials

The RIL population consisted of 206 families derived from a cross between Nongda981 (ND981) and Nongda3097 (ND3097). The population was constructed by the single seed descent method and was advanced to the F_2:6_ generation. ND981 is a cultivar characterized by lower grain weight and fewer grains per spike but more spikes; however, ND3097 has higher grain weight and more grains per spike, but fewer spikes.

A RIL family numbered RIL74 (F_6_) exhibited the residual heterozygous genotype within the mapping interval of the QTL cluster on chromosome arm 4BS and was self-pollinated for two generations (F_7_ and F_8_) to screen the recombinants. The NIL families (F_7:8_) were constructed by self-pollinated for homozygotes screened from the F_7_ population. Homozygous non-recombinant NIL families were used to evaluate the additive effect of the QTL cluster on SN, GNS, TGW, and PH, whereas homozygous recombinant NIL families and F_8_ plants were used for narrowing the candidate interval of the QTL cluster.

### Field Experiments and Traits Measurement

The RIL population (F_2:6_, F_2:7_, and F_2:8_ generations) and the two parents were sown in the autumn of 2014, 2015, and 2016 across three different geographical locations in northern China: Beijing, Handan, and Yangling (Supplementary Table 1). Each field experiment was conducted in randomized complete block design with three biological replications. Each RIL family was sown in a single row 1 m long with row spacing of 0.2 m, the planting quantity was 60 grains per row. The F_7_ and F_8_ plants were planted in rows 2 m long and 0.25 m apart at a sowing rate of 40 grains per row at Shangzhuang Town, Beijing during the 2018–2019 and 2019–2020 growing seasons, respectively. The planting method of NIL families including non-recombinant and recombinant was the same as RIL families, and they were sown at Shangzhuang Town, Beijing and Handan, Hebei Province during the 2019–2020 crop season. Water and fertilizer were managed according to the local cultivation practices.

For RILs and NIL families, three representative plants per genotype from each replication were used for measurement of PH at maturity. The survey method for SN involved taking 0.5 m per line and measuring the number of spikes along that length before harvesting. The number of spikes in 1 m rows ranged from 58 to 347. Thirty spikes of each line were randomly selected and threshed at harvesting. TGW was determined using Wanshen SC-G seed detector (Hangzhou Wanshen Detection Technology Co., Ltd.), and it was recorded as *W*_*TGW*_. The total weight of the grains, obtained from the 30 spikes, was measured using an electronic balance and recorded as *W*_*T*_. GNS was calculated by the formula as follows: *V_GNS_* = 1,000*W_*T*_/30*W_*TGW*_. For F_7_ and F_8_ plants, spike number per plant (SNPP) and height per plant (HPP) were measured at harvesting. The whole grains of each plant after threshing were used to measure the thousand grain weight of individual plant (TGWIP) and grain number per spike of individual plant (GNSIP).

### Statistical Analysis

Basic statistical analysis, phenotypic correlation, and Shapiro–Wilk tests for departure from normality were performed by SPSS software version 21.0 (SPSS, Chicago, United States). Analysis of variance (ANOVA) and broad-sense heritability ($H{}_{B}^{2}$) was conducted using QTL IciMapping software version 4.0 (Meng et al., 2015) with AOV functionality for phenotype data from seven environments for each trait. Broad-sense heritability was calculated following the formula: $H{}_{B}^{2}$ = σ*^2^_*g*_*/(σ*^2^_*g*_* + σ*^2^_*ge*_*/*n* + σ*^2^_*e*_*/*nr*), where σ*^2^_*g*_* is the genetic variance, σ*^2^_*ge*_* is the interaction variance between genotype and environment, σ*^2^_*e*_* is the residual error variance, *n* is the number of environments, and *r* is the number of replicates. The best linear unbiased prediction (BLUP) for four traits across seven environments was calculated using SAS v9.2 (SAS Institute Inc., North Carolina, United States) with the PROC MIXED procedure. In the progeny test, the phenotypic difference analysis between two genotypes was conducted by Student’s *t-*test.

### Genotyping and Construction of Genetic Map

The etiolated young leaves of the two parents and RIL population were used to extract the whole genomic DNA using the modified CTAB method (Cheng et al., 2015). The Illumina iSelect 90K wheat SNP array (Wang et al., 2014) was used to genotype the two parents and RIL population, and the genotyping was completed by the Beijing Compass Biotechnology Co., Ltd. The polymorphic SNP markers between the two parents were filtered with segregation distortion, missing, and redundancy by BIN functionality of QTL IciMapping software version 4.0 (Meng et al., 2015). After removing the low-quality SNP markers, the remaining were used to construct a genetic map using MAP functionality of QTL IciMapping software version 4.0. The maximum likelihood principle and Kosambi’s function (Kosambi, 1943) were used to determine marker order and distance, respectively. The flanking sequences of unique SNP markers were aligned to the Chinese Spring reference genome IWGSC RefSeq v1.0^1^. The best hit was taken as the physical location of each unique SNP marker. In addition, genetic linkage maps were drawn using MapChart software version 2.32 (Voorrips, 2002).

### Quantitative Trait Loci Detection

Mean values of each trait for individual environments and the adjusted mean values (BLUP) across seven environments were used for QTL mapping with BIP functionality of QTL IciMapping software version 4.0 through inclusive composite interval mapping (ICIM). The ICIM-ADD mapping method was used for determining the position and additive effects of QTL, with a scan step size of 1 cM, and LOD threshold at the default value of 2.5. The positions of QTL on chromosomes were the peak of likelihood ratios, where the LOD values exceeded the threshold of 2.5. QTL of each trait with overlapping confidence intervals (± 2 LOD away from the peak of likelihood ratios) were considered equivalent and assigned a common QTL name. QTL were named according to the International Rules of Genetic Nomenclature^2^. QTLs that could be detected in more than three individual environments and BLUP analysis were defined as stable QTLs in this work.

### InDel Markers Development

According to the resequencing data of the two parents, the insertion/deletion loci in the QTL cluster mapping interval were mined using the varTable functionality of SnpHub (Wang et al., 2020) developed by Wheat Genetics and Genomics Center of China Agricultural University. For details, please refer to following the website: https://esctrionsit.github.io/snphub_tutorial/content/Basic_Usage/vartable.html. The sequences with 200 bp on both sides of insertion/deletion loci were used to design InDel markers through Primer 3 Web version 4.1.0^3^. The PCR reaction system included 2 μl DNA template (50–200 ng/μl), 2 μl mixture of left and right primer (5 μmol/L), 5 μl 2 × Taq PCR StarMix, and 1 μl ddH_2_O. The PCR reaction procedure was set as follows: 94°C for 5 min; 34 cycles of 94°C denaturations for 30 s, 55°C–58°C annealing for 35 s, and 72°C extending for 40 s; and finally, 72°C for 10 min. The fragment length polymorphism of InDel markers was identified using 8% non-denatured polyacrylamide gel electrophoresis (PAGE). The InDel markers used in this work are listed in Supplementary Table 8.

### Candidate Genes Prediction

Gene lists in the fine mapping region of the QTL cluster were downloaded from the website: https://urgi.versailles.inrae.fr/jbrowseiwgsc/gmod_jbrowse/. Meanwhile, homologous gene IDs and functional annotations of these genes in rice were obtained^4^ and used to predict the candidate genes for SN, GNS, TGW, and PH.

## Results

### Phenotypic Performance

**FIGURE 1:**
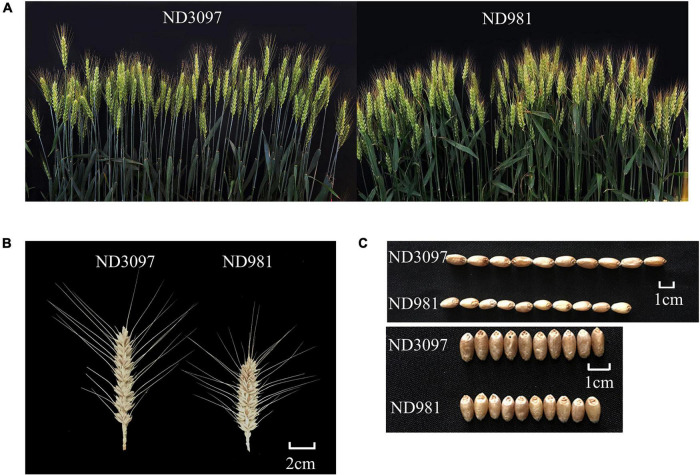
Phenotypic characterization of the two parents. **(A)** Comparison of spike numbers between ND981 and ND3097 with the single row plot of 0.2 m^2^. **(B)** Comparisons of spike size between ND981 and ND3097. Bars, 2 cm. **(C)** Comparisons of grains size between ND981 and ND3097. Bars, 1 cm.

**FIGURE 2:**
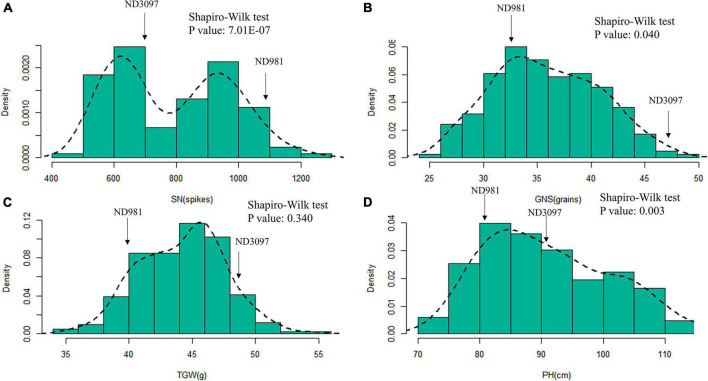
Histograms of frequency distributions of the BLUP values of three components of yield and plant height in the RIL population. **(A–D)** Histograms of frequency distributions of the BLUP values of spike number per unit area (SN), grain number per spike (GNS), thousand grain weight (TGW), and plant height (PH), respectively. The arrows represent the position of two parents for each trait in RIL population.

The means and ranges of four traits (SN, GNS, TGW, and PH) of RIL population and two parents across seven individual environments are shown in Supplementary Table 2. ND981 had higher SN and lower GNS, TGW, and PH than ND3097 in all seven environments (Figure 1 and Supplementary Table 2). The Shapiro–Wilk test and Pearson’s correlation coefficients were conducted based on BLUP values. SN displayed an obvious bimodal pattern suggesting major genes controlling it; GNS and PH displayed obvious deviations from normality, whereas TGW exhibited normal distribution (Figure 2 and Supplementary Table 2). The transgressive segregation of four traits was observed in the RIL population, suggesting that the two parents all harbored the increasing alleles of SN, GNS, TGW, and PH (Figure 2). The Pearson’s correlation coefficients of four traits showed that SN was significantly and negatively corrected with GNS and TGW, but positively corrected with PH (Supplementary Table 3). PH had a strongly positive correlation with TGW and a significantly negative correlation with GNS, whereas, the correlations between GNS and TGW were not significant (Supplementary Table 3). ANOVA across seven environments for the four traits revealed that there were significant differences among RIL genotypes (Supplementary Table 4). Broad-sense heritability of the four traits (SN, GNS, TGW, and PH) were 92.51, 93.92, 90.05, and 95.35%, respectively (Supplementary Table 4), suggesting that genetic factors were the main source of phenotypic variation of each trait.

### Genetic Map Construction

To construct a high-density genetic map covering the whole wheat genome, two parents and the RIL population were genotyped by the wheat 90K SNP array. A total of 10,384 SNP markers showed polymorphisms between ND981 and ND3097. After removing the SNP markers with more than 20% missing data and segregation distortion in the RIL population, the remaining 7,393 SNP markers, representing 1,732 unique loci, were used for linkage analysis. Finally, a high-density genetic map that included 27 linkage groups representing 21 chromosomes and covered 4,707.39 cM in length with an average density of 2.72 cM/locus was constructed for QTL mapping (Supplementary Figure 1 and Supplementary Table 5). Chromosomes 2D, 3D, 4B, 5A, 5D, and 6D were all integrated by two linkage groups (Supplementary Figure 1). The number of SNP markers distributed on three A, B, and D subgenomes was 2,298; 4,620; and 475, respectively (Supplementary Table 5).

### Quantitative Trait Loci Analysis

**FIGURE 3:**
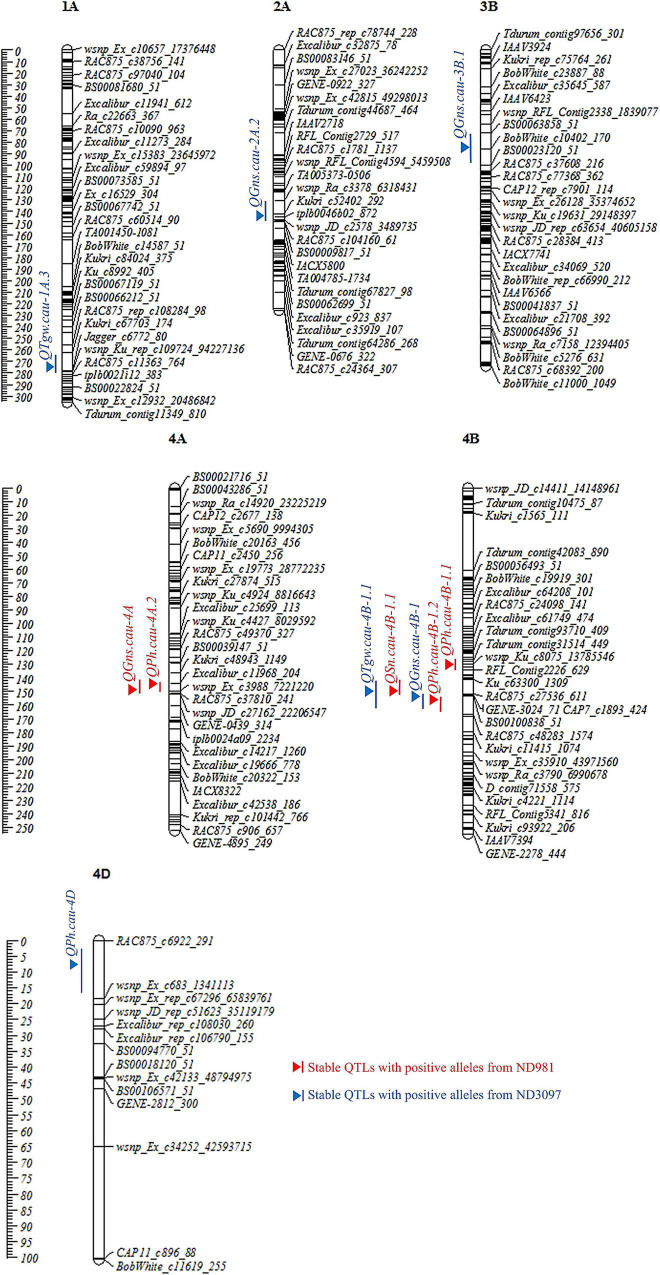
Chromosome distributions and genetic positions of stable QTL controlling spike number per unit area (SN), grain number per spike (GNS), thousand grain weight (TGW), and plant height (PH). Centimorgan (cM) scales are shown on the left. Vertical bars and triangles represent the confidence interval and LOD peak position of each QTL based on BLUP values, respectively. Red and blue represent stable QTL with favorable alleles from ND981 and ND3097, respectively.

A total of 56 QTLs were detected for SN, GNS, TGW, and PH in seven individual environments, while 19 QTLs were identified in BLUP analysis across seven environments (Supplementary Tables 6, 7). They were located on 19 chromosomes, except for chromosomes 5D and 7B. Eleven of these QTLs that could be detected in more than three individual environments and BLUP analysis were defined as stable QTLs, and the remaining 49 were putative QTLs (Figure 3, Table 1, and Supplementary Tables 6, 7). Among these QTLs, four stable and major QTL controlling SN, GNS, TGW, and PH, respectively, were detected in the same region on chromosome 4B.

**TABLE 1:** The genomic regions harboring environmentally stable QTL for spike number per unit area (SN), grain number per spike (GNS), thousand grain weight (TGW), and plant height (PH) in the ND981/ND3097 RIL population.

Chromosome	Genetic interval (cM)*^a^*	Physical interval (Mb)*^b^*	Included stable QTL*^c^*	Source of favorable allele	Phenotypic variation explained (%)	Detected environments*^d^*	References
1A	271.5–281.5	558.3–566.7	*QTgw.cau-1A.3*	ND3097	4.58–6.58	E1/E3/E7/B	
2A	125.5–147.5	723.5–735.6	*QGns.cau-2A.2*	ND3097	2.97–5.83	E1/E5/E6/E7/B	Cui et al., 2014
3B	68.5–97.5	100.9–133.8	*QGns.cau-3B.1*	ND3097	2.92–7.42	E1/E3/E5/E6/E7/B	
4A	137.5–151.5	664.1–679.3	*QGns.cau-4A*	ND981	3.78–9.93	E1/E2/E3/E4/E5/E6/E7/B	Cui et al., 2017
							Gao et al., 2015
							Guan et al., 2018
			*QPh.cau-4A.2*	ND981	2.73–4.26	E2/E4/E5/E6/E7/B	
4B-1	126.5–168.5	19.7–38.2	*QSn.cau-4B-1.1*	ND981	30.97–59.00	E1/E2/E3/E4/E5/E6/E7/B	Guan et al., 2018
							Kumar et al., 2016
							Liu et al., 2014
							Li et al., 2020
			*QGns.cau-4B-1*	ND3097	20.24–50.43	E1/E2/E3/E4/E5/E6/E7/B	Xu et al., 2019
			*QTgw.cau-4B-1.1*	ND3097	3.92–18.75	E2/E4/E6/E7/B	Chen et al., 2020
							Guan et al., 2018
							Kumar et al., 2016
							Liu et al., 2014
			*QPh.cau-4B-1.1*	ND981	3.78–5.78	E1/E2/E3/E6/B	
			*QPh.cau-4B-1.2*	ND981	11.46–28.00	E1/E3/E4/E5/E7/B	Gao et al., 2015
							Guan et al., 2018
							Wu et al., 2010
4D	0.0–11.5	13.8–54.4	*QPh.cau-4D*	ND3097	18.53–39.15	E1/E2/E3/E4/E5/E6/E7/B	Gao et al., 2015
							Guan et al., 2018
							Liu et al., 2014

*^a^The integrated confidence interval across all the detected environments.*

*^b^The corresponding physical interval (Mb) of the QTL regions were obtained by blasting the flanking sequences of SNP markers to the Chinese Spring RefSeq v1.0.*

*^c^Stable QTL were identified in above three individual environments and BLUP analysis.*

*^d^E1, 2014–2015 Handan, Hebei Province; E2, 2014–2015 Shangzhuang Town, Beijing; E3, 2014–2015 Shanxi, Yangling; E4, 2015–2016 Handan, Hebei Province; E5, 2015–2016 Shangzhuang Town, Beijing; E6, 2015–2016 Beijing, Chinese Academy of Agricultural Sciences (CAAS); E7, 2016–2017 Shangzhuang Town, Beijing. B indicates the combined QTL analysis based on BLUP values.*

#### Spike Number Per Unit Area

A total of nine QTLs for SN was identified in seven individual environments and BLUP analysis with a LOD score range of 2.52–61.24, explaining 1.48–59.00% of phenotypic variation (Supplementary Tables 6, 7). Of these QTLs, a stable and a major QTL on the short arm of chromosome 4B, *QSn.cau-4B-1.1*, was repeatedly detected in all of the seven environments as well as BLUP value, explaining the largest phenotypic variation (30.97–59.00%), and ND981 contributed the increasing allele (Figure 3, Table 1, and Supplementary Table 6).

#### Grain Number Per Spike

For GNS, a total of 16 QTLs were detected in seven individual environments and BLUP analysis with a LOD score range of 2.57–41.56, explaining 2.48–50.43% of the phenotypic variance (Supplementary Tables 6, 7). Of these QTLs, four stable QTLs were mapped on chromosomes 2A, 3B, 4A, and 4B, of which the QTL on chromosomes 4A (*QGns.cau-4A*) and 4B (*QGns.cau-4B-1*) were most stable and had a major effect, contributing up to 9.93 and 50.43% of phenotypic variation, respectively (Figure 3, Table 1, and Supplementary Table 6). The phenotypic variation explained by the remaining two stable QTLs (*QGns.cau-2A.2* and *QGns.cau-3B.1*) was 5.83 and 4.90%, respectively in the analysis of BLUP data (Figure 3 and Supplementary Table 6). The QTL *QGns.cau-4B-1* was colocated with the stable and major QTL for SN on chromosome arm 4BS (*QSn.cau-4B-1.1*), and the beneficial allele was from ND3097 (Figure 3 and Table 1).

#### Thousand Grain Weight

For TGW, a total of 21 QTLs were detected in seven individual environments and BLUP analysis with a LOD score range of 2.55–30.89, explaining 1.13–27.09% of the variance (Supplementary Tables 6, 7). Of these QTLs, two stable QTLs were identified on chromosomes 1A (*QTgw.cau-1A.3*) and 4B (*QTgw.cau-4B-1.1*), and the alleles for increased TGW at two loci were contributed by ND3097. The QTL *QTgw.cau-4B-1.1* was colocated with the major stable QTL for SN and GNS (*QSn.cau-4B-1.1* and *QGns.cau-4B-1*) and explained 3.92–18.75% of the phenotypic variation for TGW (Figure 3, Table 1, and Supplementary Table 6).

#### Plant Height

For PH, a total of 14 QTLs were detected in seven individual environments and BLUP analysis with a LOD score range of 2.52–27.74, explaining 2.24–39.15% of phenotypic variation (Supplementary Tables 6, 7). Of these QTLs, four stable QTLs were located on chromosomes 4A (*QPh.cau-4A.2*), 4B (*QPh.cau-4B-1.1* and *QPh.cau-4B-1.2*) and 4D (*QPh.cau-4D*), the increasing alleles of *QPh.cau-4A.2*, *QPh.cau-4B-1.1*, and *QPh.cau-4B-1.2* all were from ND981, whereas ND3097 contributed to the increasing allele of the QTL *QPh.cau-4D* (Figure 3 and Supplementary Table 6). The two stable QTLs *QPh.cau-4A.2* and *QPh.cau-4B-1.1* explained relatively lower variance (2.73–4.26% and 3.78–5.78%). The mapping intervals of the two QTLs *QPh.cau-4B-1.2* and *QPh.cau-4D*, explaining the higher phenotypic variation (up to 28.00 and 39.15%), included the dwarfing gene loci *Rht-B1* and *Rht-D1*, respectively. The QTL *QPh.cau-4B-1.2* was colocated with the stable major QTL *QSn.cau-4B-1.1*, *QGns.cau-4B-1*, and *QTgw.cau-4B-1.1* (Figure 3, Table 1, and Supplementary Table 6).

### Remapping for the Quantitative Trait Loci Cluster

**FIGURE 4:**
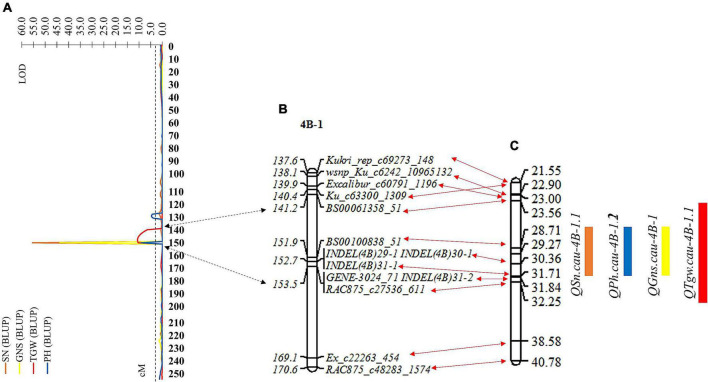
Remapping for the QTL cluster based on the resultant linkage map and BLUP values of spike number per unit area (SN), grain number per spike (GNS), thousand grain weight (TGW), and plant height (PH). **(A)** The LOD values map of SN, GNS, TGW, and PH on chromosome 4B. **(B)** Partial resultant linkage map for the genomic region of the QTL cluster. **(C)** The corresponding physical map for the genomic region of the QTL cluster. The double-headed arrows represent the corresponding position between **(A–C)**. Black and red indicate the confidence interval of the QTL cluster corresponding to the position of resultant linkage map and partial resultant linkage map corresponding to the position of the physical map, respectively. The rectangles represent the confidence interval of the QTL. Orange, blue, yellow, and red indicate SN, GNS, TGW, and PH, respectively.

The genomic region of the chromosome arm 4BS flanked by SNP markers *Ku_c63300_1309* and *RAC875_c48283_1574* was identified and considered a QTL cluster controlling four stable and major QTLs for SN, GNS, TGW, and PH in the RIL population. To further dissect the identified QTL cluster consisting of QTL *QSn.cau-4B-1.1*, *QGns.cau-4B-1*, *QTgw.cau-4B-1.1*, and *QPh.cau-4B-1.2*, we developed four polymorphic InDel markers within the overlapping region of these four QTLs to genotype the RIL population (Supplementary Table 8). These four InDel markers were added to the resultant linkage map, and the genetic distance changing from 254.93 to 257.60 cM was also covered (Figure 4B and Supplementary Table 9). Subsequently, the remapping for the QTL cluster was conducted based on the new resultant genetic map of chromosome 4B and BLUP values of SN, GNS, TGW, and PH (Figure 4A). The QTL cluster was relocated to a physical interval of about 8.7 Mb delimited by SNP markers *BS00061358_51* and *RAC875_c27536_611*, including three QTL (*QSn.cau-4B-1.1*, *QGns.cau-4B-1*, and *QPh.cau-4B-1.2*) for SN, GNS, and PH, respectively, colocated to the interval flanked by markers *BS00100838_58* and *INDEL(4B)31-1* (∼3.0 Mb), and one QTL (*QTgw.cau-4B-1.1*) for TGW delimited to the interval by markers *BS00061358_51* and *RAC875_c27536_611* (∼8.7 Mb) (Figure 4C). The four QTLs *QSn.cau-4B-1.1*, *QGns.cau-4B-1*, *QPh.cau-4B-1.2*, and *QTgw.cau-4B-1.1* explained 70.47, 54.02, 18.58, and 8.79% of phenotypic variation for SN, GNS, PH, and TGW, respectively.

### Fine Mapping of the Quantitative Trait Loci Cluster

**FIGURE 5:**
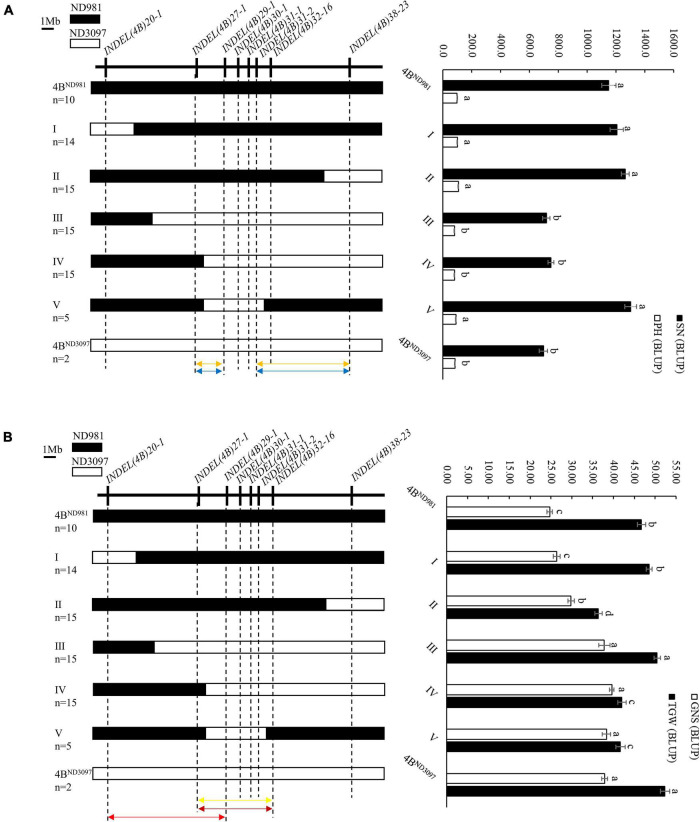
Fine mapping of the QTL cluster using the homozygous recombinant F_7:8_ families. **(A)** Fine mapping of the *QSn.cau-4B-1.1* and *QPh.cau-4B-1.2*. **(B)** Fine mapping of the *QGns.cau-4B-1* and *QTgw.cau-4B-1.1*. *Left side* is the eight InDel markers used to screen homozygous recombinants (*upside*) and the graphical genotypes of five homozygous recombination types and two parent types (*downside*). n represents the number of each homozygous family. Black and white bars represent the chromosome segments from ND981 and ND3097, respectively. Orange arrows represent the interval of fine mapping for *QSn.cau-4B-1.1*, blue arrows represent the interval of fine mapping for *QPh.cau-4B-1.2*, yellow arrow represents the interval of fine mapping for *QGns.cau-4B-1*, carmine arrow represents the interval of fine mapping for *QTgw.cau-4B-1.1^ND981^*, red arrow represents the interval of fine mapping for *QTgw.cau-4B-1.1^ND3097^*. *Right side* is the comparisons of spike number per unit area (SN), grain number per spike (GNS), thousand grain weight (TGW) and plant height (PH) among each homozygous family. The values of SN, GNS, TGW and PH are the means (mean ± SE) of each homozygous family. a, b, c and d represent the significance of differences between different homozygous families.

The RHL is one of the main and efficient methods for fine-mapping of QTL without extensive backcrossing, especially for self-pollinated crops (Liu N. et al., 2018; Chen et al., 2020). In this study, one RHL (RIL74) displaying heterozygous genotype within the mapping interval of the QTL cluster on chromosome arm 4BS was screened from the F_6_ generation of the RIL population and used for fine mapping of the QTL cluster. To validate the additive effect of the QTL cluster and narrow its candidate interval, a total of eight polymorphic InDel markers covered the wider region of the QTL cluster for 20.0–38.0 Mb of chromosome 4B were used for genotyping F_7_ population generated from self-pollinated of RIL74 (Supplementary Table 8). Finally, 43 homozygous non-recombinant F_7_ plants representing two parental types and 64 homozygous recombinant F_7_ plants representing five recombination types were identified and planted to generate NIL families, F_7:8_. Among them, 43 homozygous non-recombinant F_7:8_ families, namely 4B^ND981^ NILs (with ND981 allele) and 4B^ND3097^ NILs (with ND3097 allele), were used to validate the additive effect of the QTL cluster, and on 64 homozygous recombinant F_7:8_ families, namely I, II, III, IV, and V NILs were conducted phenotypic identification to narrow the candidate interval of the QTL cluster (Figure 5).

In subsequent student’s *t-*test of the progeny, the averages SN and PH of 4B^ND981^ NILs were extremely significantly higher than that of 4B^ND3097^ NILs in both the environments (Table 2), whereas, the averages, GNS and TGW, of 4B^ND981^ NILs were significantly lower than that of 4B^ND3097^ NILs in both the environments (Table 2). These results indicated that the ND981 allele of the QTL cluster has a positive effect on SN and PH, but has a negative effect on GNS and TGW. The extremely significant phenotypic differences in SN, GNS, TGW, and PH were detected between IV and 4B^ND981^ NILs (*p* < 0.01). Moreover, besides SN and PH, the extremely significant effects for GNS and TGW were observed between V and 4B^ND981^ NILs (*p* < 0.01). Remarkably, the average TGW of IV NILs was lower than that of 4B^ND3097^ NILs, and the average TGW of V NILs was lower than that of 4B^ND981^ NILs. This implied that the QTL for TGW was decomposed into two linked QTLs with opposite effects, tentatively named as *QTgw.cau-4B-1.1^ND981^* and *QTgw.cau-4B-1.1^ND3097^*. Taken together, QTL *QSn.cau-4B-1.1* and *QPh.cau-4B-1.2* were delimited into the interval flanked by markers *INDEL(4B)27-1* and *INDEL(4B)29-1* or *INDEL(4B)31-2* and *INDEL(4B)38-23; QGns.cau-4B-1* and *QTgw.cau-4B-1.1^ND981^* were colocated between markers *INDEL(4B)27-1* and *INDEL(4B)32-16*, and *QTgw.cau-4B-1.1^ND3097^* was delimited into the interval flanked by markers *INDEL(4B)20-1* and *INDEL(4B)29-1.*

**TABLE 2:** Average spike number per unit area (SN), grain number per spike (GNS), thousand grain weight (TGW), and plant height (PH) of homozygous non-recombinant F_7:8_ families in two environments.

Env.*^a^*	Genotype*^b^*	F_7:8_			
		SN	GNS	TGW	PH
E8	4B^ND981^	1,215	23.3	44.87	99.2
	4B^ND3097^	695	36.1	50.55	82.5
	Percentage	74.82%***	−35.46%***	−11.24%**	20.24%***
E9	4B^ND981^	1,090	24.7	48.56	97.6
	4B^ND3097^	700	35.6	54.20	81.5
	Percentage	55.71%***	−30.62%***	−10.41%**	19.75%***

^a^E8, 2019–2020 Shangzhuang Town, Beijing; E9, 2019–2020 Handan, Hebei Province; ^b^Positive percentage values indicate the increasing allele is from homozygous non-recombinant F_7:8_ families with ND981 allele (4B^ND981^ NILs), negative percentage values indicate the increasing allele is from homozygous non-recombinant F_7:8_ families with ND3097 allele (4B^ND3097^ NILs); **p < 0.01; ***p < 0.001.

**FIGURE 6:**
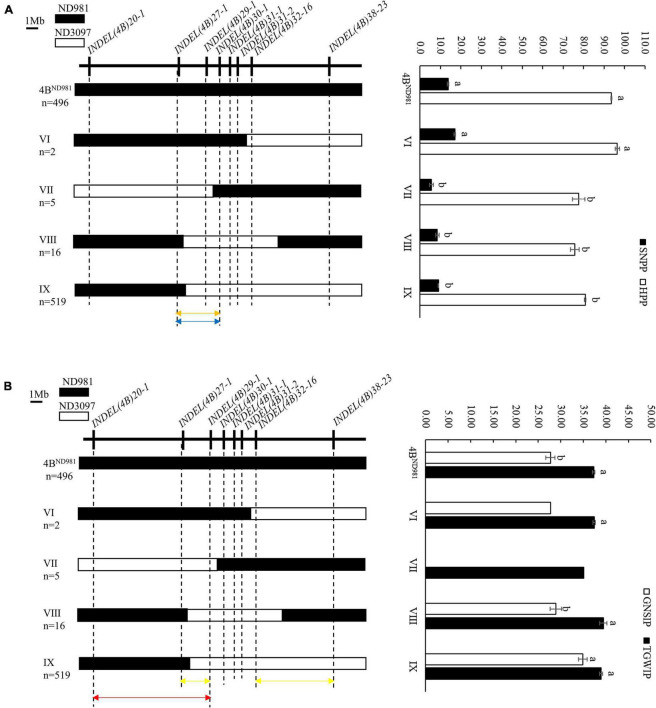
Fine mapping of the QTL cluster using the homozygous recombinant F_8_ plants. **(A)** Fine mapping of the *QSn.cau-4B-1.1* and *QPh.cau-4B-1.2*. **(B)** Fine mapping of the *QGns.cau-4B-1* and *QTgw.cau-4B-1.1*. *Left side* is the eight InDel markers used to screen homozygous recombinants (*upside*) and the graphical genotypes of four homozygous recombination types and the parent ND981 type (*downside*). n represents the number of each homozygous plant. Black and white bars represent the chromosome segments from ND981 and ND3097, respectively. Orange arrows represent the interval of fine mapping for *QSn.cau-4B-1.1*, blue arrows represent the interval of fine mapping for *QPh.cau-4B-1.2*, yellow arrow represents the interval of fine mapping for *QGns.cau-4B-1*, carmine arrow represents the interval of fine mapping for *QTgw.cau-4B-1.1^ND981^*, red arrow represents the interval of fine mapping for *QTgw.cau-4B-1.1^ND3097^*. *Right side* is the comparisons of spike number per plant (SNPP), grain number per spike of individual plant (GNSIP), thousand grain weight of individual plant (TGWIP) and height of per plant (HPP) among each homozygous plant. The values of SNPP, GNSIP, TGWIP, and HPP are the means (mean ± SE) of each homozygous plant. Here a and b represent the significance of differences between different homozygous plants.

To use the phenotypic data of individual plants to accelerate the fine mapping of the QTL cluster, the correlation analysis for average SN, GNS, TGW, and PH of F_7:8_ families and average SNPP, GNSIP, TGWIP, and HPP of corresponding F_7_ plants were performed. The results indicated that SN, GNS, TGW, and PH of F_7:8_ families all were significantly correlated with SNPP, GNSIP, TGWIP, and HPP of the corresponding F_7_ plants (*P* < 0.05), respectively, which provided good support for using SNPP, GNSI, TGWIP, and HPP of individual plants for assessing SN, GNS, TGW, and PH of their progenies in advance (Supplementary Table 10). Therefore, the F_8_ plants were screened by the eight polymorphic InDel markers used for screening F_7_ plants to further narrow the candidate region of the QTL cluster. A total of 496 homozygous non-recombinant F_8_ plants displaying ND981 genotype and 542 homozygous recombinants representing four recombination types, namely type 4B^ND981^, VI, VII, VIII, and IX, were obtained from the F_8_ population (Figure 6). After the Student’s *t-*test of progeny, average SNPP and HPP of type 4B^ND981^ F_8_ plants were extremely significantly higher than that of type VII, VIII, and IX F_8_ plants (*p* < 0.01). Average TGWIP of type VIII and IX F_8_ plants, and average GNSIP of type VIII F_8_ plants had no significant difference with type 4B^ND981^ F_8_ plants, but the average GNSIP of type IX F_8_ plants was extremely significantly higher than that of type 4B^ND981^ F_8_ plants. These results suggested that the genomic region flanked by markers *INDEL(4B)27-1* and *INDEL(4B)30-1* harbored two QTL *QSn.cau-4B-1.1* and *QPh.cau-4B-1.2* for SN and PH, respectively, and QTL *QGns.cau-4B-1* for GNS and was delimited into the interval flanked by markers *INDEL(4B)27-1* and *INDEL(4B)29-1*, or *INDEL(4B)32-16* and *INDEL(4B)38-23;* QTL *QTgw.cau-4B-1.1* for TGW was delimited into the interval flanked by markers *INDEL(4B)20-1* and *INDEL(4B)29-1*.

Combined fine-mapping results of F_7:8_ families and F_8_ plants, *QSn.cau-4B-1.1*, *QPh.cau-4B-1.2*, and *QGns.cau-4B-1* were colocated between markers *INDEL(4B)27-1* and *INDEL(4B)29-1*. *QTgw.cau-4B-1.1* was divided into two linked QTL with opposite effects, tentatively named as *QTgw.cau-4B-1.1^ND981^* and *QTgw.cau-4B-1.1^ND3097^*. The former was located between markers *INDEL(4B)27-1* and *INDEL(4B)32-16*, with the favorable allele from ND981, and the latter was located between markers *INDEL(4B)20-1* and *INDEL (4B)29-1*, with the favorable allele from ND3097.

### Prediction of Candidate Genes

According to fine mapping of the QTL cluster, the interval flanked by markers *INDEL(4B)27-1* and *INDEL(4B)29-1* was selected for candidate genes analysis. Based on Chinese Spring IWGSC RefSeq Annotations v1.1^5^, a total of 31 high confidence genes were annotated in the *INDEL(4B)27-1* and *INDEL(4B)29-1* interval (Supplementary Table 11). Among them, *TraesCS4B02G041600* may be a candidate gene for *QTgw.cau-4B-1.1*, *QGns.cau-4B-1*, and *QPh.cau-4B-1.2*, and *TraesCS4B02G042400* may be a candidate gene for *QPh.cau-4B-1.2* and *QGns.cau-4B-1*. *TraesCS4B02G041600* was predicted to encode a Mitogen-activated protein kinase 1. In recent years, several members of the gene families encoding mitogen-activated protein kinase were reported to be involved in regulating TGW, GNS, and PH in rice, for e.g., *mitogen-activated protein kinase 15* (*OsMPK15*) (Hong et al., 2019), *mitogen-activated protein kinase 4* (*OsMKK4*) (Guo T. et al., 2018), and *mitogen-activated protein kinase 6* (*OsMAPK6*) (Liu et al., 2015). The functional description of *TraesCS4B02G042400* was phosphatidylinositol 4-phosphate 5-kinase 1, which is the homologous gene of *Os03G0705300* in rice. *Os03G0705300* has been shown to affect stem elongation and panicle development in rice (Fang et al., 2020). For SN, homologous genes of these 31 high confidence genes in rice and Arabidopsis have not been reported to regulate tiller. It is necessary to further narrow the candidate interval of *QSn.cau-4B-1.1* to reduce the number of candidate genes.

## Discussion

### Comparisons of Quantitative Trait Loci for Yield-Related Traits With Previous Research

Three components of yield and PH are important agronomic traits for improving wheat yield potential. Identification of genetic loci controlling these four traits is the basis for breeding high-yield wheat varieties (Zhang et al., 2010). In this study, a total of 11 environmentally stable QTL for three components of yield (SN, GNS, and TGW) and PH were detected in the ND981/ND3097 RIL population, which were mainly distributed on chromosomes 1A, 2A, 3B, 4A, 4B, and 4D (Figure 3, Table 1, and Supplementary Table 6). Among them, the QTL *QGns.cau-2A.2*, *QGns.cau-4A* and *QGns.cau-4B-1* for GNS were located on chromosomes 2A, 4A, and 4B at the similar physical positions of QTL reported by Cui et al. (2014), Gao et al. (2015), and Xu et al. (2019), respectively. Two major stable QTLs (*QPh.cau-4B-1.2* and *QPh.cau-4D*) for PH identified on chromosomes 4B and 4D were consistent with the dwarfing genes *Rht-1* and *Rht-2* (Peng et al., 1999). The major stable QTL for SN, *QSn.cau-4B-1.1*, was detected on chromosome 4B corresponding to the QTL identified by Guan et al. (2018) and Li et al. (2020). *QTgw.cau-4B-1.1* had a similar physical position with QTL for TGW reported by the previous studies (Liu et al., 2014; Kumar et al., 2016; Guan et al., 2018; Chen et al., 2020). In addition to the above seven stable QTLs, the remaining four stable QTLs were likely novel QTLs for GNS, TGW, and PH.

### Genetic Relationship Between Three Components of Yield and PH

Three components of yield influenced each other during the growth and development of wheat, and also are affected by other traits, such as PH, crop phenology, and biomass (Guan et al., 2018). In this work, four stable and major QTLs controlling SN (*QSn.cau-4B-1.1*), GNS (*QGns.cau-4B-1*), TGW (*QTgw.cau-4B-1.1*), and PH (*QPh.cau-4B-1.2*) were identified on the same region of chromosome arm 4BS. Among them, the source of positive alleles of *QSn.cau-4B-1.1* and *QPh.cau-4B-1.2* were opposite to that of *QGns.cau-4B-1* and *QTgw.cau-4B-1.1* (Figure 2 and Supplementary Table 6), which exhibited the strong trade-off between SN, PH and GNS, TGW, consistent with phenotypic correlation analysis (Supplementary Table 3). For SN and TGW, Guan et al. (2018) has reported two stable QTLs (*QSpp.cau-4B.3* and *QTgw.cau-4B.2*) located in the physical interval of about 13.98–30.86 Mb on chromosome 4B, and we achieved similar results. Moreover, the positive alleles of *QSpp.cau-4B.3* and *QTgw.cau-4B.2* were from the opposite parent, which indicated a trade-off relationship between SN and TGW.

For PH and TGW, some studies showed that positive alleles of the colocated QTL controlling them were from the same parent, which was in contrast to this work. For example, Guan et al. (2018) detected a stable QTL (*QPh.cau-4B.2*) on chromosome 4B controlling PH with superior alleles coming from the parent JD6, consistent with the source of a favorable allele of *QTgw.cau-4B.2*, which demonstrated a positive relationship between PH and TGW. In addition, the increasing alleles of two colocated major QTLs on chromosome arm 3DS controlling PH and TGW all were contributed by the parent HS2 (Chen et al., 2020). Moreover, some studies on dwarfing genes *Rht-1* and *Rht-2* have shown that the dwarfing allele not only reduced PH, but also decreased TGW due to linkage drag (Jobson et al., 2018). In this work, two tightly linked QTLs controlling PH and TGW, *QPh.cau-4B-1.2* and *QTgw.cau-4B-1.1*, explaining the highest phenotypic variance, were identified on chromosome 4B. The positive allele of *QPh.cau-4B-1.2* was from the parent ND981, whereas that of *QTgw.cau-4B-1.1* was from another parent ND3097, which may be caused by *QTgw.cau-4B-1.1*, further divided into two linked QTLs with opposite effects, and the additive effect of *QTgw.cau-4B-1.1^ND3097^* was larger than *QTgw.cau-4B-1.1^ND981^*during the subsequent fine mapping. This result provided a theoretical basis and genetic resources for breeding new varieties of wheat with lower PH and higher grain weight.

### Breeding Value of QTgw.cau-4B-1.1

Thousand grain weight is an important agronomic trait in wheat and plays a significant and stable role in improving wheat yield (Wu et al., 2015). At present, several studies have identified stable and major QTLs for TGW near the dwarfing gene *Rht1*on chromosome 4B (Table 1), accounting for 4.75–39.30% of the phenotypic variance. In this work, we also identified a major QTL for TGW (*QTgw.cau-4B-1.1*) spanning the dwarfing gene *Rht1* using the ND981/ND3097 RIL population, the favorable allele coming from the parent ND3097 with higher grain weight. *QTgw.cau-4B-1.1* was divided into two linked QTLs with opposite effects in subsequent fine-mapping using secondary segregation populations derived from the RIL74 family, tentatively named as *QTgw.cau-4B-1.1^ND981^* and *QTgw.cau-4B-1.1^ND3097^*. The additive effect of *QTgw.cau-4B-1.1^ND3097^* was larger than that of *QTgw.cau-4B-1.1^ND981^*, which was in accordance with the source of a favorable allele of *QTgw.cau-4B-1.1* in primary mapping. The additive effect analysis showed that the average TGW of F_7:8_ families carrying favorable allele of *QTgw.cau-4B-1.1^ND3097^* was increased by 10.51 g than carrying unfavorable allele of *QTgw.cau-4B-1.1^ND3097^*, whereas *QTgw.cau-4B-1.1^ND981^* was increased 4.85 g. Therefore, the combination of a favorable allele of these two QTL identified in this study may significantly increase TGW and thus improve wheat yield, which is of great value for breeding the high-yield wheat varieties.

## Conclusion

Overall, a total of 60 QTLs for SN, GNS, TGW, and PH were identified on 19 chromosomes except for chromosomes 5D and 7B in the ND981/ND3097 RIL population across seven environments and BLUP values. Among them, four major and stable QTLs for SN, GNS, TGW, and PH were located on the same genomic region of chromosome 4B, which was considered a QTL cluster. Further, the fine mapping results showed that three QTLs (*QSn.cau-4B-1.1*, *QGns.cau*-*4B-1*, and *QPh.cau-4B-1.2*) were colocated to the physical interval of about 1.4 Mb, and *QTgw.cau-4B-1.1* was located to a physical interval of about 9.3 Mb and divided into two linked QTLs (*QTgw.cau-4B-1.1^ND981^* and *QTgw.cau-4B-1.1^ND3097^*) with opposite effects. Based on homologous genes of rice within the candidate interval, *TraesCS4B02G041600* was considered a candidate gene for *QTgw.cau-4B-1.1*, *QGns.cau-4B-1*, and *QPh.cau-4B-1.2*, and *TraesCS4B02G042400* also may be a candidate gene for *QPh.cau-4B-1.2* and *QGns.cau-4B-1*.

## Data Availability Statement

The original contributions presented in the study are included in the article/Supplementary Material, further inquiries can be directed to the corresponding author/s.

## Author Contributions

MY conceived the project. SW carried out the experiments and analyzed the experimental results and wrote the original draft. ST, CB, HZ, CW, ZC, JY, and XS participated in the field trials. MY, QS, MZ, and CF helped to review and revise the original draft. KT helped with the production of figures in the manuscript. All authors have read and approved the final manuscript.

## Conflict of Interest

The authors declare that the research was conducted in the absence of any commercial or financial relationships that could be construed as a potential conflict of interest.

## Publisher’s Note

All claims expressed in this article are solely those of the authors and do not necessarily represent those of their affiliated organizations, or those of the publisher, the editors and the reviewers. Any product that may be evaluated in this article, or claim that may be made by its manufacturer, is not guaranteed or endorsed by the publisher.

## Footnotes

1
https://urgi.versailles.inra.fr/blast_iwgsc/blast.php

2
https://wheat.pw.usda.gov/ggpages/wgc/98/Intro.htm

3
https://primer3.ut.ee/

4
http://wheat.cau.edu.cn/TGT/

5
https://urgi.versailles.inra.fr/download/iwgsc/IWGSC_RefSeq_Annotations/v1.1/
